# White Light Emission from Vegetable Extracts

**DOI:** 10.1038/srep11118

**Published:** 2015-06-17

**Authors:** Vikram Singh, Ashok K. Mishra

**Affiliations:** 1 Department of Chemistry, Indian Institute of Technology Madras, Chennai, 600036 India

## Abstract

**A mixture of extracts from two common vegetables, red pomegranate and turmeric, when photoexcited at 380** **nm, produced almost pure white light emission (WLE) with Commission Internationale d’Eclairage (CIE) chromaticity index (0.35, 0.33) in acidic ethanol. It was also possible to obtain WLE in polyvinyl alcohol film (0.32, 0.25), and in gelatin gel (0.26, 0.33) using the same extract mixture. The colour temperature of the WLE was conveniently tunable by simply adjusting the concentrations of the component emitters. The primary emitting pigments responsible for contributing to WLE were polyphenols and anthocyanins from pomegranate, and curcumin from turmeric. It was observed that a cascade of Forster resonance energy transfer involving polyphenolics, curcumin and anthocyanins played a crucial role in obtaining a CIE index close to pure white light. The optimized methods of extraction of the two primary emitting pigments from their corresponding plant sources are simple, cheap and fairly green.**

White light emitting materials have attracted significant attention in recent years as key components in display and lighting devices based on LEDs^1^. LEDs accounts for almost 20% of the total worldwide energy consumption and have wide applications in backlights, displays, lasers and indicators^2^,^3^,^4^. White light emission has also been used for sensing^5^. There has been a high level of interest in recent years in looking for white light emitting organic and inorganic molecules and materials, when photoexcited at near UV wavelengths^6^. A variety of photophysical principles have been used for achieving emission containing the three essential red-green-blue components for effective white light perception. These include FRET^7^,^8^, Inter- and intra- molecular charge transfer^9^, excited state intramolecular proton transfer (ESIPT)^10^, hydrogen bonding mediated J- aggregation^11^ and the mixing of monomer and excimer fluorescence^12^ etc. Although single-molecule WLE is conceptually attractive, it is difficult to adjust the CIE index and colour temperature of such molecules conveniently. In this regard multi component WLE comprising of a mixture of different molecules can, in principle, make it easy to tune the colour temperature by simply adjusting the composition. Not surprisingly, there is significant recent interest for generation of mixed emitter WLE. Such systems comprise of inorganic, organic and hybrid systems like nanomaterials/quantum dots^13^,^14^,^15^, polymer^16^,^17^, metal-organic framework^18^,^19^,^20^, inorganic-organic hybrids^21^, metal complexes^22^ and lanthanide doped systems^23^,^24^. Various systems tried by using mixtures of organic fluorophores include, (i) a simple mixing of three emitting dyes at RGB region^25^, (ii) donar-acceptor conjugated pairs^26^, (iii) organic liquids such as π- conjugated oligo (p-phenylenevinylene) (OPV)^27^, (iv) controlled donor self assembly based organogels^28^, (v) and a peptide link based two component system^29^.

Interestingly, generation of white light emission from natural dyes has not been looked at yet. Plant sources have been rich sources of colouring dyes since time immemorial and many of such dyes are strongly fluorescent. *Thus, while looking for white light emitters, it makes sense to look for a combination of suitable natural dyes.* Literature search did not yield any such report in this regard. We report here the result of our search for a white light emitting natural dye combination based on the following criteria: (i) the sources of the dyes must be easily available and cheap, (ii) the extraction of the dyes must be simple, cost effective and environment friendly, (iii) the dyes must have sufficient high extinction coefficient and fluorescence quantum yield so that the combination has good white light emission efficiency, (iv) the individual dye emissions must be suitably located in the CIE plot so as to give white light emission after mixing, and (v) the colour temperature of the emission should be conveniently tunable by simply adjusting the dye composition. In order to achieve facile tuning of CIE index as well as the colour temperature of interest, we followed a strategy in choosing two vegetable extracts containing emitting dyes such that they emit in blue-violet, green and orange regions of visible spectrum.

For this purpose, several trials with blue, green and red emitting natural dyes^30^ eventually converged on to two easily available vegetable extracts, red pomegranate (Pom) seed juice and turmeric (Tur) extract. The essential emitting pigments in Pom seed juice are a group of polyphenolics and anthocyanins^31^. In general, polyphenols and anthocyanins emit at blue and orange-red region respectively^32^,^33^ and are found in many of the fruits and vegetables^34^. Curcumin is the essential emitting pigment in Tur extract^35^. The molecular structures of the natural derivatives are shown in Fig. 1a,b respectively.

Pom is rich in antioxidants such as phenolic acids, tannins, flavonols, and anthocyanins^36^. The red colour of Pom is known to be primarily due to six anthocyanin pigments. Hernández *et al*. have quantitatively and qualitatively analyzed all six anthocyanins by high-performance liquid chromatography and identified them as delphinidin 3- glucoside and 3, 5-diglucoside, cyanidin 3-glucoside and 3, 5-diglucoside and pelargonidin 3-glucoside and 3, 5-diglucoside^37^. The colour of anthocyanins is pH sensitive; in acidic media it shows a red colour, while in alkaline solutions, it turns blue^38^.

There are various methods and sources reported in literature for extraction of polyphenols and anthocyanins such as pink petals of rhododendron indicum flowers^32^, Pom^39^,^40^, blackcurrant^41^, grapes^42^, red cabbage^43^,etc. We have adopted a variant of a method reported by Hamidreza *et al.*^37^, the details of which are given in the section on Materials and Methods.

## Results and discussion

Electronic absorption and fluorescence spectral studies were carried out in the extraction medium containing 1% HCl in ethanol. For fluorescence spectra, the excitation wavelength was fixed at 380 nm. UV-visible electronic spectra (black) of Pom and Tur extracts are shown in Fig. 2a and Fig. 2b respectively. The strong absorption band at 535 nm originates from anthocyanins and at ~295 nm from the polyphenolic components^31^,^32^,^44^. The total anthocyanin concentration was estimated using the known molar extinction coefficient (34300 M^−1^cm^−1^) of cyanindin-3-glucoside at 530 nm^45^. Since the amount of emitting natural dyes can change from source to source, the effective concentration of the dyes can be expressed in terms of molarity calculated with this molar extinction coefficient. This practice has been followed in the rest of this paper.

For the Tur extract, a broad characteristic^46^ absorption band of curcumin is seen around 350–500 nm with maximum at 420 nm (Fig. 2b (black)). This absorption corresponding to π – π* electronic transition has molar extinction coefficient of 55000 M^−1^ cm^−1^ at 420 nm in ethanol^43^. The effective concentration of the emitting dye in the Tur extract has subsequently been expressed in terms of molarity calculated with this extinction coefficient. Under UV light excitation at 380 nm, Pom extract shows bluish red emission (inset, Fig. 2a) (blue) with two bands at 440 nm and 590 nm corresponding to the phenolic and anthocyanin components respectively^32^,^33^,^44^. For the same excitation Tur extract shows intense green fluorescence emission (green) at around 522 nm^47^ (Fig. 2b).

The CIE chromaticity^48^ coordinate for Pom extract appears in the blue-violet region A(0.28, 0.17); and the coordinate for Tur extract appears at green region B(0.31, 0.51) in the CIE diagram (Fig. 3). It is observed that the points A and B appear on the opposite side of the white region in the CIE diagram. This suggested to us that by mixing the two extracts it should be possible to obtain WLE.

**Figure 1:**
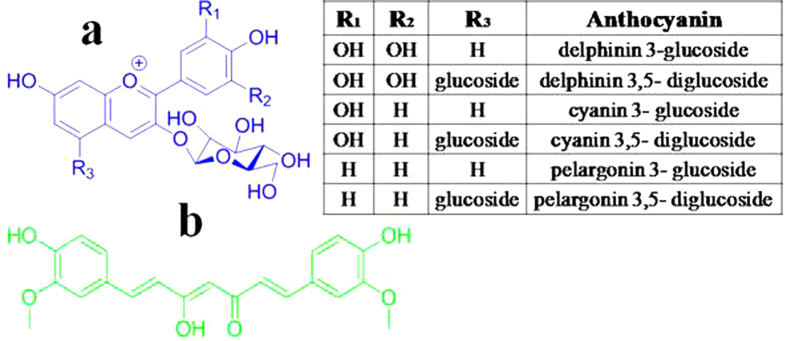
Chemical Structure. (**a**) Anthocyanins and (**b**) Curcumin.

**Figure 2:**
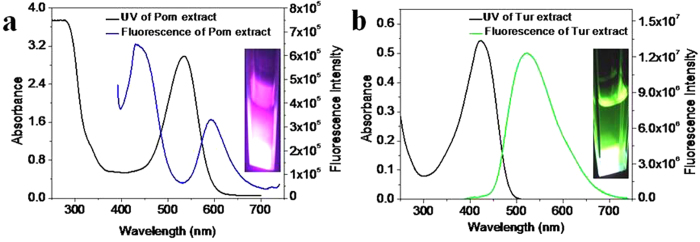
UV-Visible spectra (black) and fluorescence spectra. (**a**) Pom extract (blue line), where [anthocyanin] = 85 μM and (**b**) Tur extract (green line), where [Curcumin] = 9.85 μM with their fluorescent images under uv excitation, in 1% HCl ethanol. [λ_exc_ = 380 nm].

Accordingly, a series of solutions were prepared by varying the relative concentrations of Pom and Tur extracts (Supplementary Table S1, Fig 1a and Fig. 1b). Figure 4 shows the trajectory of the colour coordinate of these solutions in the CIE diagram. It is seen that the point C (0.35, 0.33), corresponding to the composition 60.0 μM (anthocyanin equivalent) and 3.0 μM (curcumin equivalent) with a molar ratio of 1:0.05::anthocyanin:curcumin, is extremely close to the pure white coordinate O (0.33, 0.33).

It is interesting to see that the trajectory of the colour coordinate actually bends and passes very close to the pure white coordinate. The emission spectrum corresponding to point C covered the entire visible region (400–700 nm) (Fig. 5a) with two emission maxima at 480 and 590 nm. These two bands, though appear to be similar to polyphenolic and anthocyanin emissions of Pom, have different characteristics. For the extract mixture, there is a red shift of blue emission band from 440 nm to 480 nm and a significant enhancement as well as broadening of the 590 nm band. Clearly, this change in the emission spectral features is due to contribution from Tur emission. The excitation emission matrix fluorescence (Fig. 5b) clearly shows that at 380 nm excitation the emission covers the entire visible spectral range upto about 700 nm.

**Figure 3:**
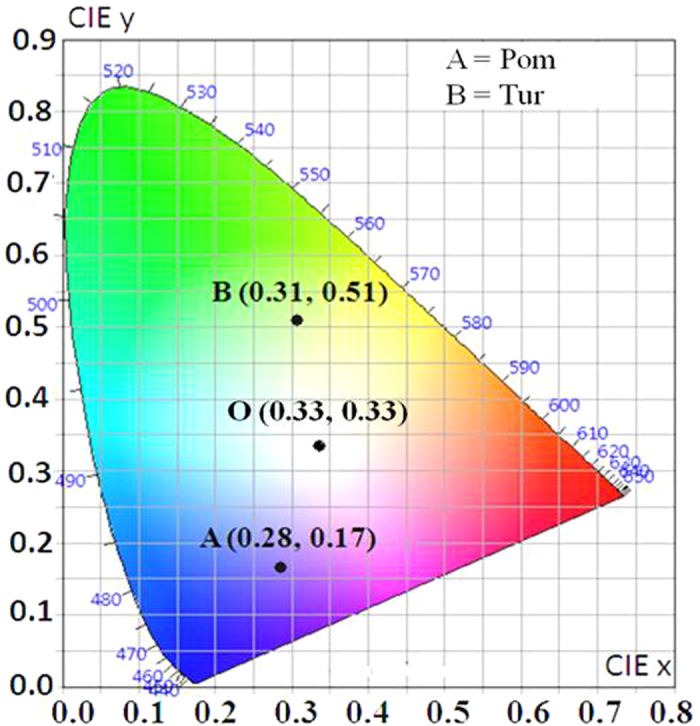
CIE-1931 diagram. Chromaticity plot for colour coordinates of Pom (**A**) and Tur (**B**) extracts. The point O indicates pure white light coordinates.

**Figure 4:**
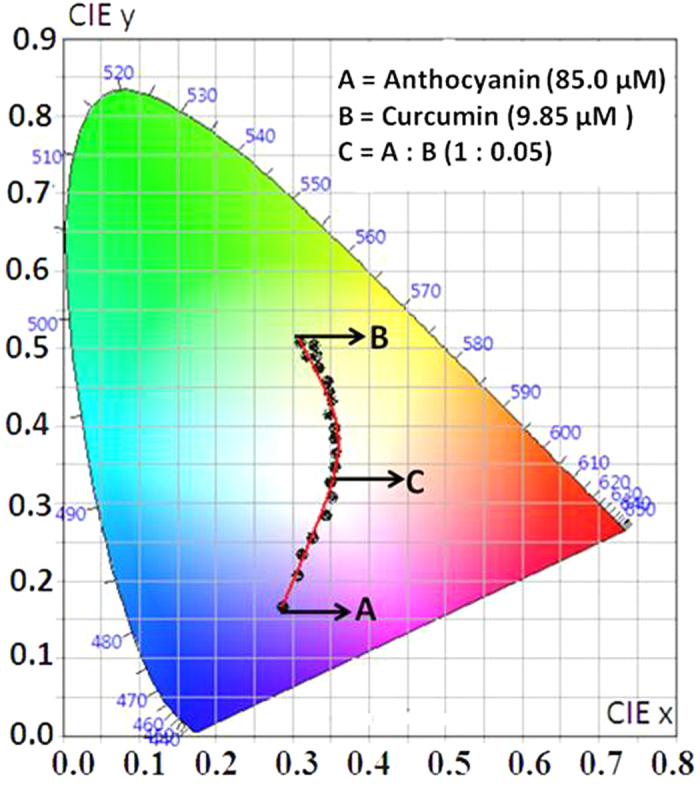
CIE-1931 diagram. Chromaticity plot for colour coordinates after mixing of Pom extract with Tur extract in acidic ethanol solution.

**Figure 5:**
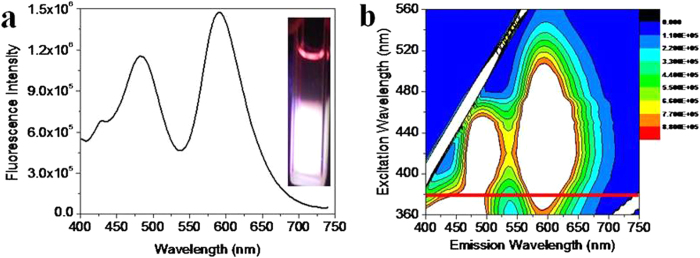
WLE in acidic ethanol. (**a**) Emission spectrum of a mixture of Pom extract (anthocyanin equivalent 60.0 μM) and Tur extract (curcumin equivalent 3.0 μM), excited at 380 nm. Inset shows photograph of corresponding white emitting solution under UV excitation (380 nm) and (**b**) Excitation emission matrix fluorescence spectrum of white emitting solution at different excitation wavelength (λ_exc_ = 360–560 nm).

**Figure 6:**
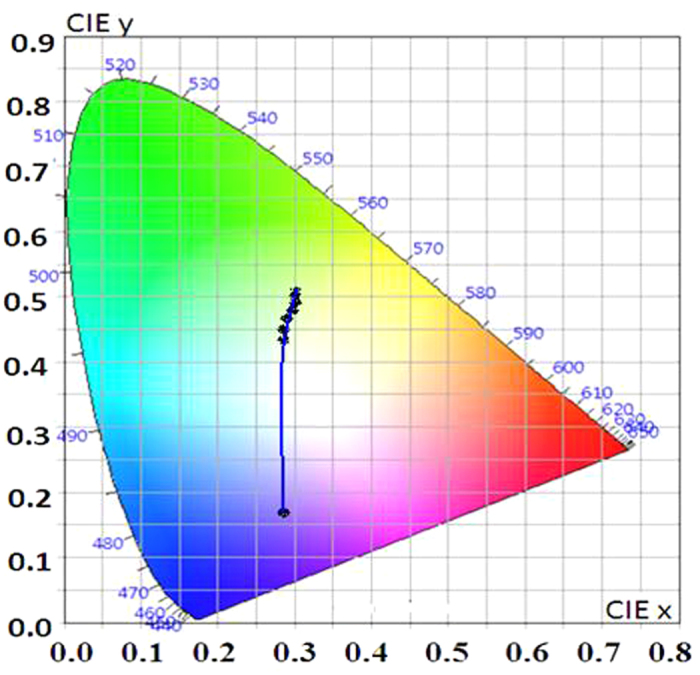
CIE-1931 diagram. Chromaticity for the projected fluorescence emission spectra after addition of individual emissions of Pom and Tur extracts.

If the components in the two extracts did not interact photophysically, their emission spectral profile would be additive. Such hypothetical emission spectra (Supplementary Fig. S3) can be created by adding the intensities of component spectral profiles of Pom and Tur extracts (Supplementary Fig. S2). The corresponding colour coordinates of these sum spectra in the CIE diagram are shown in Fig. 6. The reported quantum yield of malvidin 3, 5-diglucoside anthocyanin^49^ is 4.1 × 10^−3^ (generally, the anthocyanins are weakly fluorescent) and that of curcumin^50^ is 6.3 × 10^−2^. Hence it is seen that the Tur extract fluorescence is much more intense than the Pom extract fluorescence. A comparison of the trajectory, the hypothetical sum spectral coordinates (Fig. 6) and the actual spectral coordinate (Fig. 3) indicates the possible presence of significant photophysical interaction between the components.

**Figure 7:**
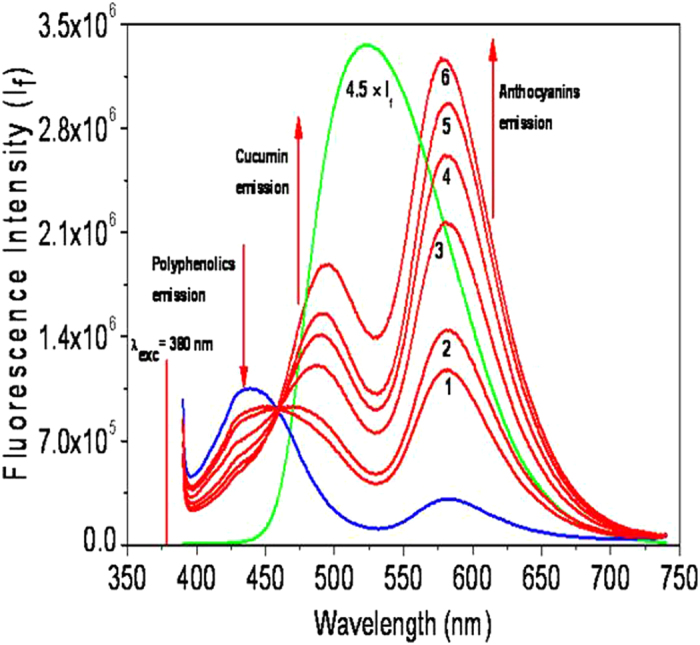
The FRET Cascade. 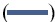
 Polyphenolics emission band at 440 nm and 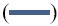 anthocyanin emission band at 585 nm, 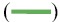 curcumin emission band at 522 nm, 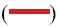 Changes of emission bands (1-6) with progressive addition of Tur extract (1-6 μM).

The absorption and emission spectra of the two extracts show significant overlap (Supplementary Fig. S4) of (i) curcumin absorption band (420 nm) with polyphenolics emission band (440 nm), and (ii) curcumin emission band (522 nm) with anthocyanin absorption band (535 nm). Thus there is a strong possibility of Forster resonance energy transfer (FRET) cascading from polyphenolics to curcumin to anthocyanins. In ordered to examine the presence of FRET, changes in spectral profiles were monitored with progressive addition of Tur extract (0–6 µM) to a fixed amount of Pom extract (50 µM) (Fig. 7).

As is seen in the Fig. 7, with progressive increase of the Tur extract component, there is progressive (i) decrease of the polyphenolics emission around 440 nm, (ii) increase of curcumin emission around 490 nm and (iii) increase of anthocyanins emission around 585 nm. These spectral changes can be ascribed to a FRET cascade from polyphenolics to curcumin to anthocyanins because of the following observations: (i) Significant spectral overlap of emission band of polyphenolics with the absorption band of curcumin, and the emission band of curcumin with the absorption band of anthocyanins, thereby satisfying the essential spectral overlap condition for FRET (Supplementary Fig. S4), (ii) the loss of polyphenolics emission, (iii) the significant loss of curcumin emission intensity in the mixture, (iv) the substantial enhancement of the long wavelength emission at the expense of curcumin emission and (v) the interesting observation that there is a dip of the curcumin emission at 530 nm which is the absorption maximum of anthocyanin absorption.

Observations from a complimentary experiment in which increasing amount Pom extract was added to a fixed Tur extract, given in (Supplementary Fig. S5) shows the progressive loss of curcumin emission intensity with a dip at 530 nm corresponding to maximum spectral overlap of anthocyanin absorption and curcumin emission. This further supports the existence of FRET. The presence of the FRET cascade in the optimized mixture of Pom and Tur extracts thus results in an extended emission spectrum that covers the entire visible spectral range from 400 nm to 700 nm. It also explains the bending of the trajectory towards red thereby making it pass through the pure white CIE. The concept of FRET has been further explored for adjusting the colour temperature. The ‘colour temperature’ of emission from a source refers to the temperature (in Kelvin unit) at which the colour of black body radiation matches the colour of the emissive source. The CIE coordinates and colour temperatures of a few combinations of Pom and Tur in acidic ethanol have been given in table 1. As is seen in table 1, simple variations in relative composition of the components shift the CIE indices and the corresponding colour temperatures in a facile way.

**Figure 8:**
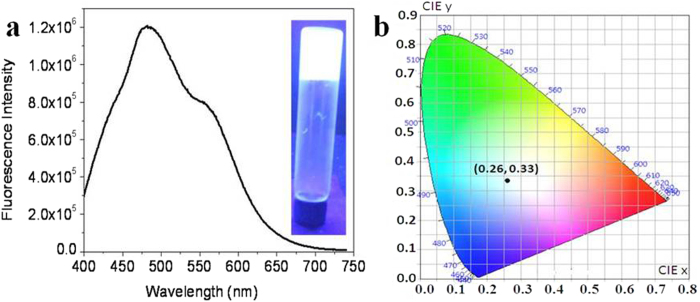
WLE in gelatin gel. (**a**) Emission spectrum of white light mixture with gelatin gel in acidic ethanol and water (46 : 54%) at excitation 380 nm, inset shows pure white photograph of gelatin gel under UV excitation and (**b**) CIE plot for colour coordinate of white light emitting gelatin gel (0.26, 0.33).

The possibility of WLE using Pom and Tur extracts in media like gelatin gel and polyvinyl alcohol (PVA) film has also been explored. Gelatin is an edible, biodegradable and biocompatible polymer that is produced by the thermal or physical and chemical degradation of collagen. WLE mixture was mixed with prepared gelatin solution and was kept in refrigerator for forming the gel. The resulting solution in acidic ethanol and water (46:54%) forms (Supplementary Fig. 6a) white light emitting gel at 15 °C under UV light illumination. Fluorescence spectrum of gel has been shown in Fig. 8a, which covered the visible region from 400 to 650 nm. This gel shows good white light emission under UV light (inset, Fig. 8a). The CIE colour coordinate for WLE mixed gelatin gel was found to be (0.26, 0.33) (Fig. 8b).

**Figure 9:**
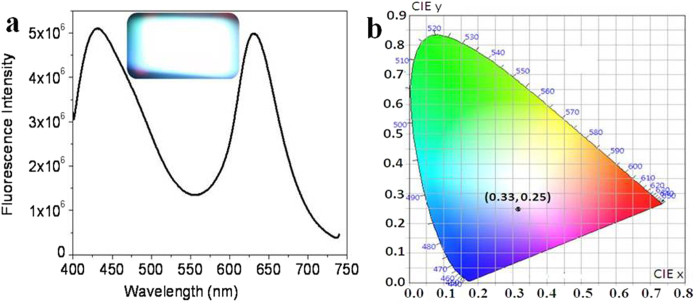
WLE in PVA film. (**a**) Emission spectrum of WLE incorporated PVA film, inset shows excellent white photograph of PVA film under UV excitation (380 nm) and (**b**) CIE plot for colour coordinate of white light emitting PVA film (0.32, 0.25).

The prepared dry PVA film was kept with WLE mixture in water-ethanol (10:90) for swelling overnight. Subsequently, the swollen film was allowed to dry (Supplementary Fig. 6b). Fluorescence emission spectrum of dye incorporated PVA film shows two bands at 450 and 625 nm (Fig. 9a). A good CIE coordinate value (0.32, 0.25) was obtained from the corresponding emission spectrum (Fig. 9b).

## Conclusions

In summary, we have generated white light emission from natural dyes extracted in our laboratory using a green and simple procedure. The optimized mixture of two suitably chosen plant extracts using acidic ethanol, aided by a FRET cascade from polyphenolics to curcumin to anthocyanins, generates almost pure white light, with CIE values of (0.35, 0.33) in solution, (0.26, 0.33) in gelatin gel and (0.33, 0.25) in PVA film. White light emission from such cheap and nature friendly resources could be important in the context of lighting and sensing application. It would be interesting to see if such system can be used as dyes for tunable dye laser applications. To the best of our knowledge this is the first time low cost, biocompatible (edible) natural dyes have been a part of white light emitting system. Given the vast number of excellent natural fluorescent dyes obtainable from renewable biosources, approaches similar to the present could lead to a more extensive range of low-cost and efficient WLE biomaterials with ease of adjusting colour temperature, which will obviate more expensive alternatives currently being pursued.

## Materials and methods

### Materials and Instruments

Pom, Tur and gelatine gel were purchased from local market of Chennai, India. Spectroscopic grade solvents were purchased from Sigma-Aldrich for extraction of dyes from natural resources. Ploy vinyl alcohol (PVA) (m.wt. approx 150000) was also purchased from Sigma-Aldrich for film preparation. UV-VIS electronic absorption spectra measurements were carried out in a Jasco V-650 UV-Visible Spectrophotometer with a scan rate of 1000 nm s^−1^. Fluorescence emission spectra of the samples were measured using Fluoromax-4 (Horiba Jobin Yvon) spectrofluorometer, with a xenon lamp of 150 W as excitation source. Excitation and emission monochromator band passes were kept at 5 nm and quartz cell cuvette (1 × 1 cm). CIE colour coordinates have been calculated using freely available online *Osram* Sylvania software^51^.

### Method for Dye Extraction

Red Pom seeds were squeezed and the extract centrifuged at 5000 rpm. As reported^37^, the clear supernatant contained polyphenols and anthocyanins as major dye components. The extraction of Curcumin from *Curcuma longa* root (Tur) was carried out by grinding the rhizome with mortar and pestle using ethanol as extracting solvent. This extract was centrifugated for 5 minutes at 5000 rpm to obtain a clear yellow coloured supernatant that contained Curcumin as the major dye. These optimized methods of extraction are fairly simple and environment friendly.

### Polymeric Film and Gel Preparation

#### Preparation of PVA Film

PVA dry film was prepared by dissolving 1 g in 10 ml of distilled water (10% w/v) at 60 °C. This solution was poured into flat-bottomed dishes in such a manner that a uniform thin layer of liquid covers the surface. This dish was kept in the oven at 80 °C and film was allowed to dry. The dry polymer film could be easily detached from the dish.

#### Preparation of Gelatin Solution

Gelatin gel was prepared by dissolving 1 g in 20 ml of distilled water (5% w/v) with constant stirring at 65 °C. This solution, when mixed with the dye extracts and set at 15 °C, forms the white emitting gel.

## Additional Information

**How to cite this article**: Singh, V. and Mishra, A. K. White Light Emission from Vegetable Extracts. *Sci. Rep.* **5**, 11118; doi: 10.1038/srep11118 (2015).

## Supplementary Material

Supplementary Information

## Figures and Tables

**Table 1:**
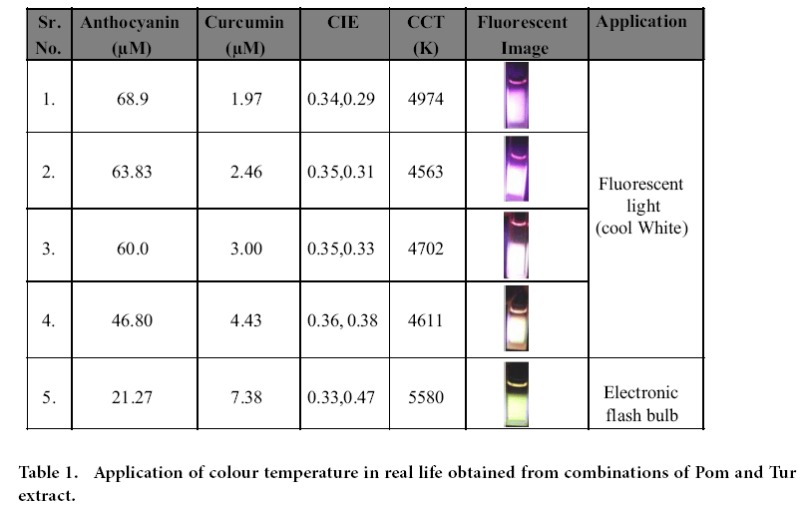
Application of colour temperature in real life obtained from combinations of Pom and Tur extract.
