# Supplementary material for: White Light Emission from Vegetable Extracts
Source: Sci Rep. 2015 Jun 17;5:11118. doi: 10.1038/srep11118 (PMC4470329; doi:10.1038/srep11118)
Supplement: Supplementary Information [file srep11118-s1.pdf]

Supporting Information

White Light Emission from Vegetable Extracts

Vikram Singh and Ashok K. Mishra*

Department of Chemistry, Indian Institute of Technology Madras,

Chennai, 600036 India

Correspondence E-mail: mishra@iitm.ac.in

Supplementary Table 1| Various concentration combinations of Pom with Tur (A-U) for obtaining colour temperature

Sr. No.	Anthocyanin (μM)	Curcumin (μM)	CIE	CCT (K)	Application	
A	85.0	0	0.28,0.17	>16000	sky blue	clear sky blue
B	81.14	0.49	0.31,0.21	15387		clear sky blue
C	76.60	0.98	0.32,0.23	7870		cloudy sky
D	72.34	1.47	0.33,0.26	5683	sunlight	noon
E	68.09	1.97	0.34,0.29	4974	fluorescent light	cool white
F	63.83	2.46	0.35,0.31	4563		cool white
**G**	**60.00**	**3.00**	**0.35,0.33**	**4702**		**cool white**
H	55.32	3.44	0.36,0.34	4373		cool white
I	51.07	3.94	0.36,0.36	4505		cool white
J	46.80	4.43	0.36,0.38	4611		cool white
K	42.56	4.92	0.36,0.40	4697		cool white
L	38.29	5.41	0.35,0.41	5009		cool white
M	34.04	5.91	0.35,0.43	5054		cool white
N	29.78	6.40	0.34,0.44	5323	electronic flash bulb	
O	25.53	6.89	0.34,0.46	5340		
P	21.27	7.38	0.33,0.47	5580		
Q	17.02	7.88	0.33,0.49	5578		
R	12.76	8.37	0.32,0.49	5801	sunlight	
S	8.51	8.86	0.32,0.50	5792		
T	4.24	9.35	0.32,0.50	5792		
U	0	9.85	0.31,0.51	5998		

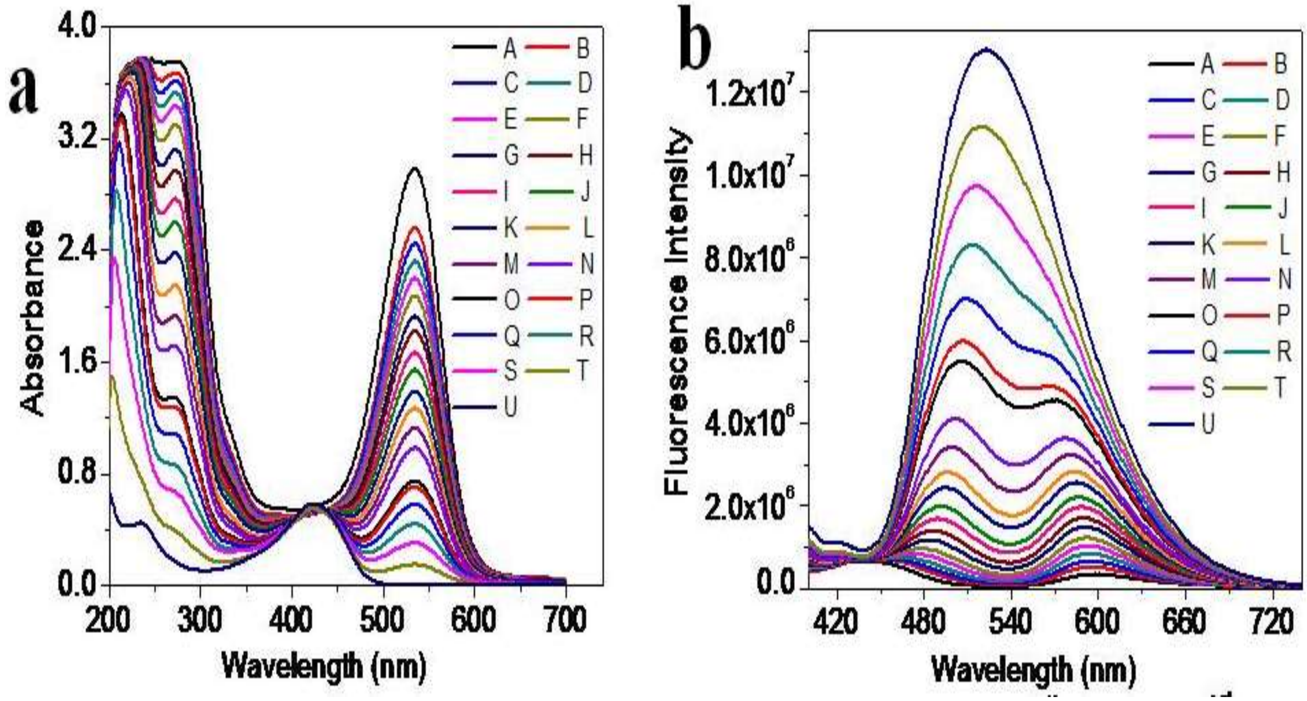

Supplementary Figure 1|Observed UV-Visible and fluorescence spectra at various concentration combinations of Pom and Tur extract. (a) UV-Visible electronic spectra and **(b)** Fluorescence spectra on mixing of Pom and Tur extract in different concentration in 1% HCl ethanol [λ_{exc}= 380 nm].

Detail of concentration combinations (A-U) have been given above in Table 1

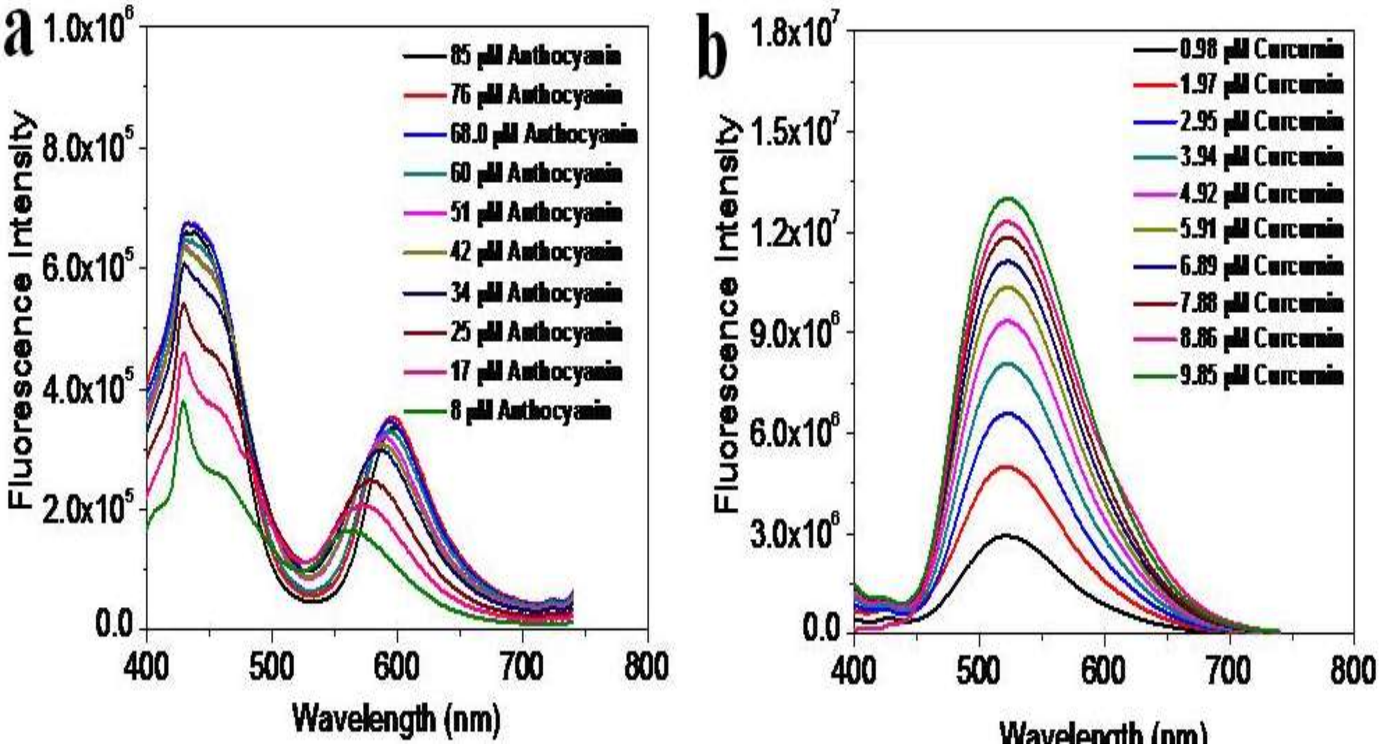

Supplementary Figure 2|Fluorescence Spectra of two vegetable extract. (a) Pom extrcat, where [anthocyanin] = 7 μM – 66 μM and **(b)** Tur extract, where [curcumin] = 0.98 μM – 9.85 μM at various concentrations in 1% HCl ethanol [λ_{exc} = 380 nm].

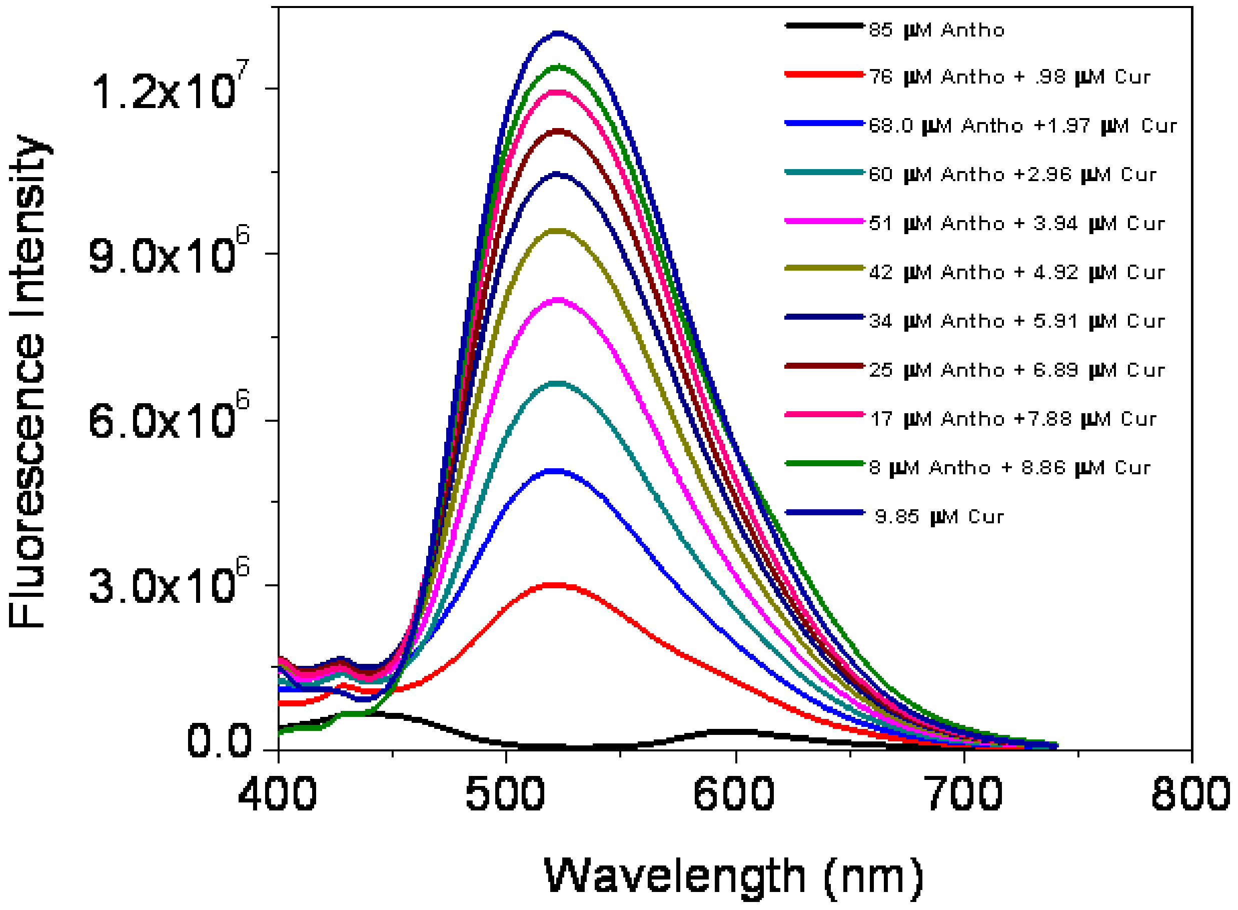

Supplementary Figure 3 | Projected Fluorescence Spectra. Hypothetically additive Fluorescence spectra of Pom and Tur extract in 1% HCl ethanol [λ_{exc} = 380 nm].

Where, Antho = Anthocyanin and Cur = curcumin

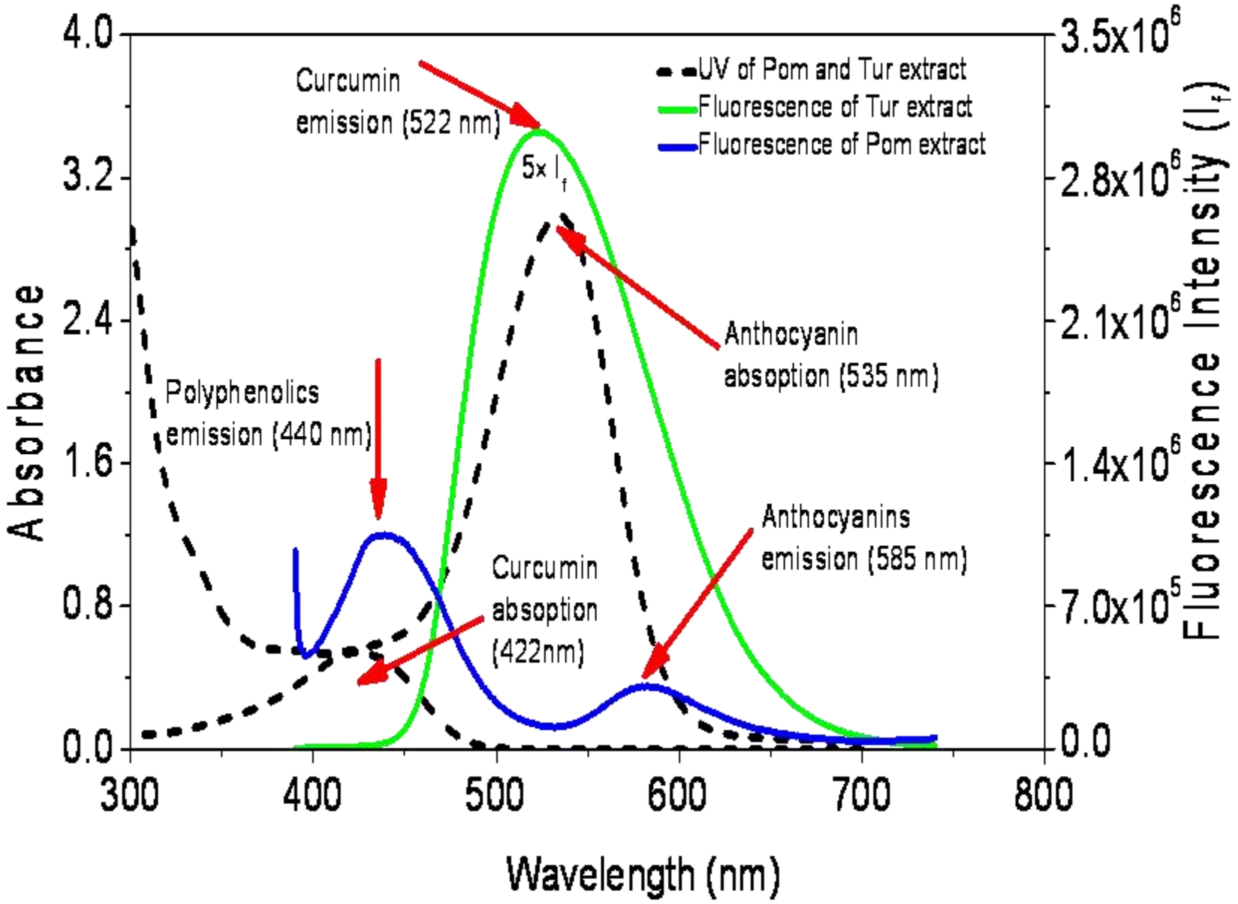

Supplementary Figure 4|Spectral overlap of absorption and emission bands. (i) Emission band of Polyphenols (blue) with absorption band of Tur extract (--- black) are showing perfect overlapping (ii) Fluorescence spectrum of Tur (green) with absorption bnad of anthocyanin (--- black) are showing perfect overlapping in 1% HCl ethanol [λ_{exc}= 380 nm].

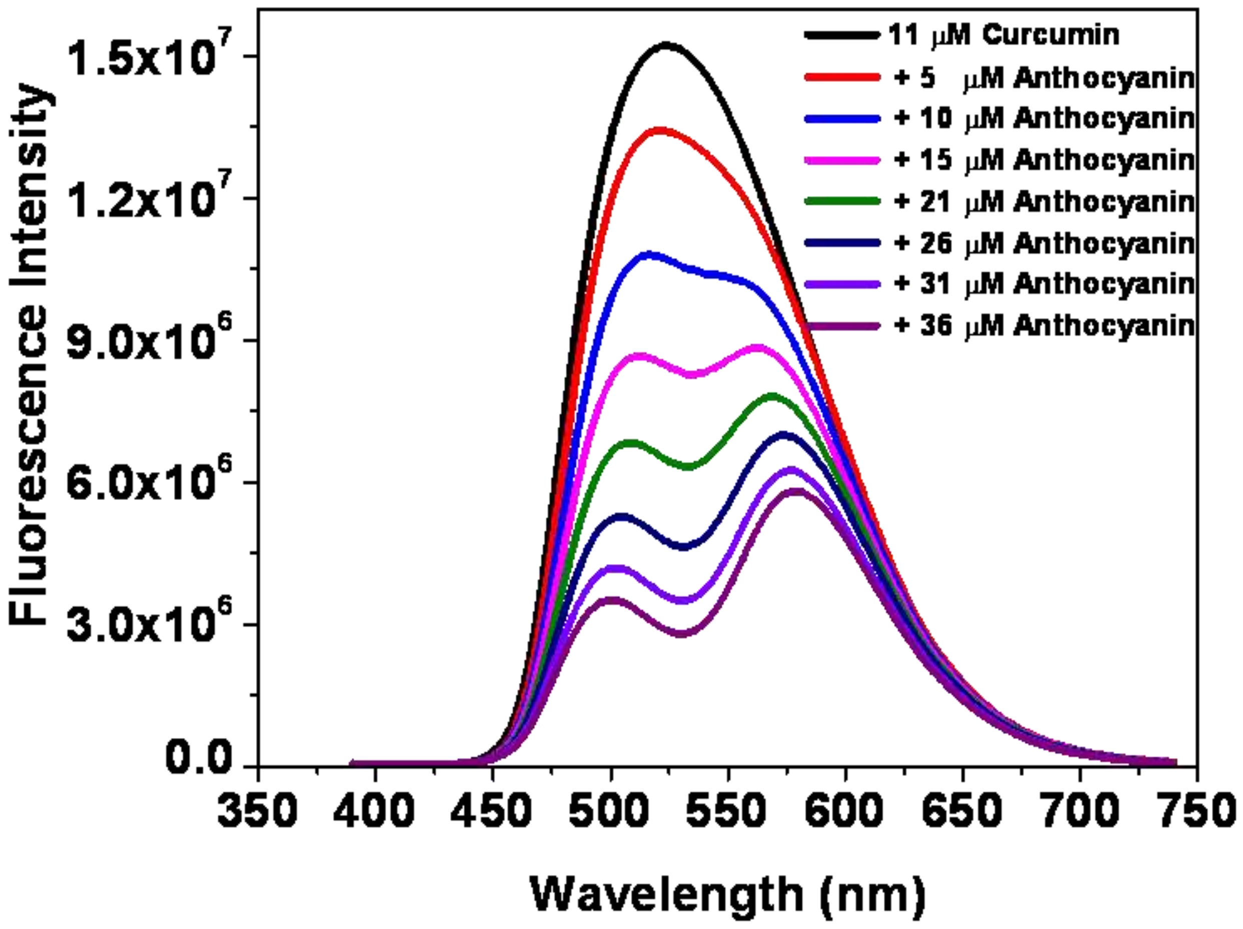

Supplementary Figure 5|Change in Fluorescence spectrum of Tur (11 μM).

On gradual addition of Pom (5-36 μM) in 1% HCl ethanol [λ_{exc}= 380 nm]

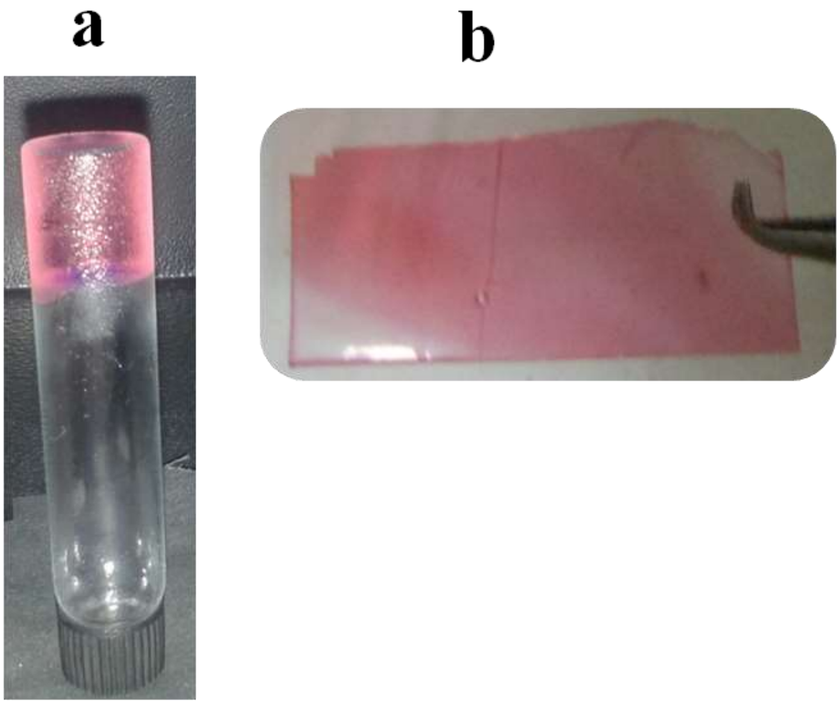

Supplementary Figure 6|Photographs of gelatin gel and PVA film under white light. (a) Image of gelatin gel mixed with WLE solution and **(b)** WLE solution incorporated PVA film.
